# Supplementary material for: High-Level Production of Soluble Cross-Reacting Material 197 in Escherichia coli Cytoplasm Due to Fine Tuning of the Target Gene’s mRNA Structure
Source: BioTech (Basel). 2023 Jan 11;12(1):9. doi: 10.3390/biotech12010009 (PMC9844443; doi:10.3390/biotech12010009)
Supplement: Supplementary file 1 [file biotech-12-00009-s001.zip › biotech-2074539-supplementary.pdf]

SUPPLEMENTARY INFORMATION

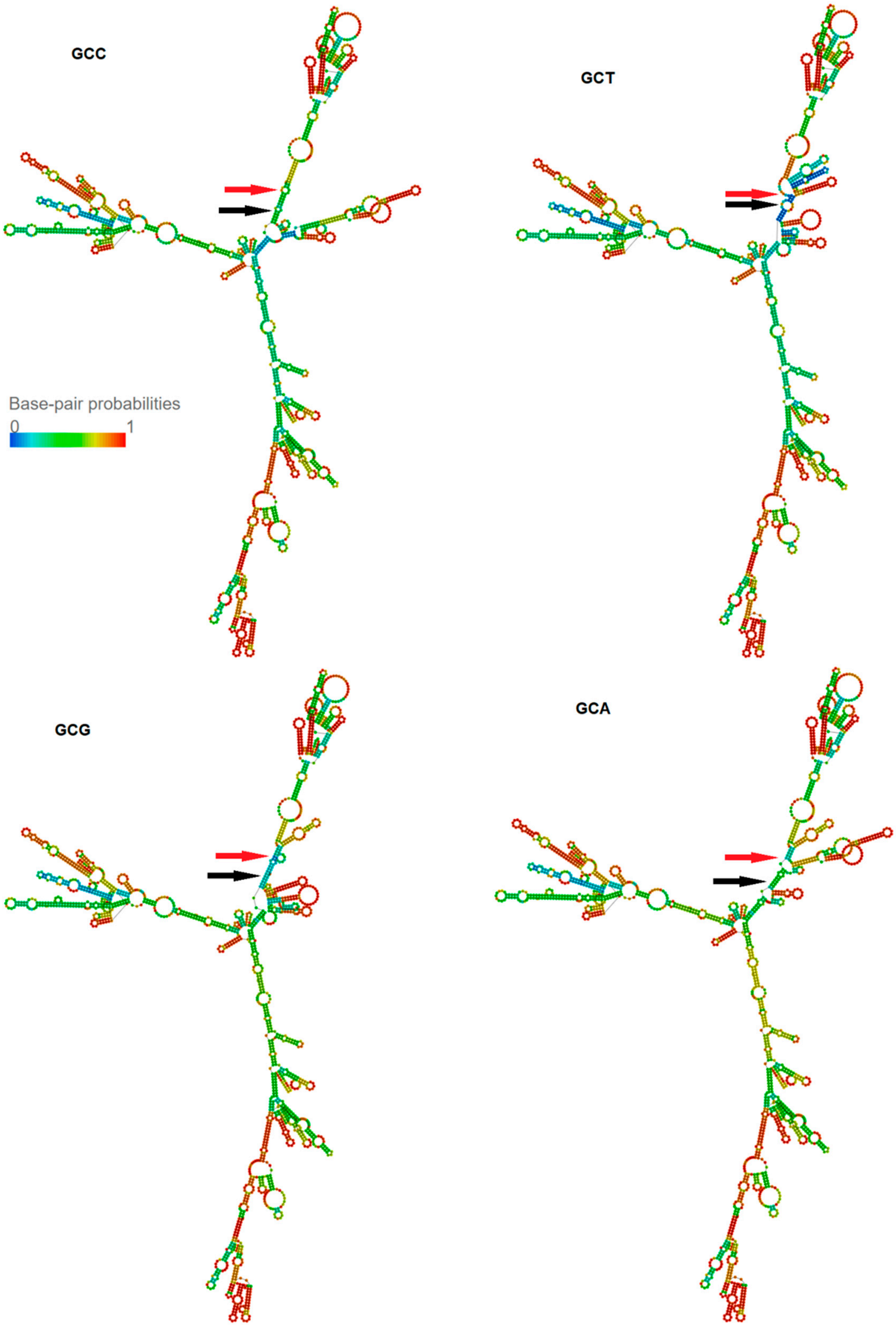

Figure S1. Secondary structures of the CRM197-encoding mRNA variants predicted using RNAfold software. The sequence from the supposed transcription start to the supposed transcription terminator (1825 bases) was taken for the analysis. The mutated codon is written near each structure; its position in the mRNA sequence is shown with a black arrow. The AUG start codon is shown with a red arrow. The predicted probability of being paired or unpaired is color-coded from blue (zero probability) to red (100 % probability).

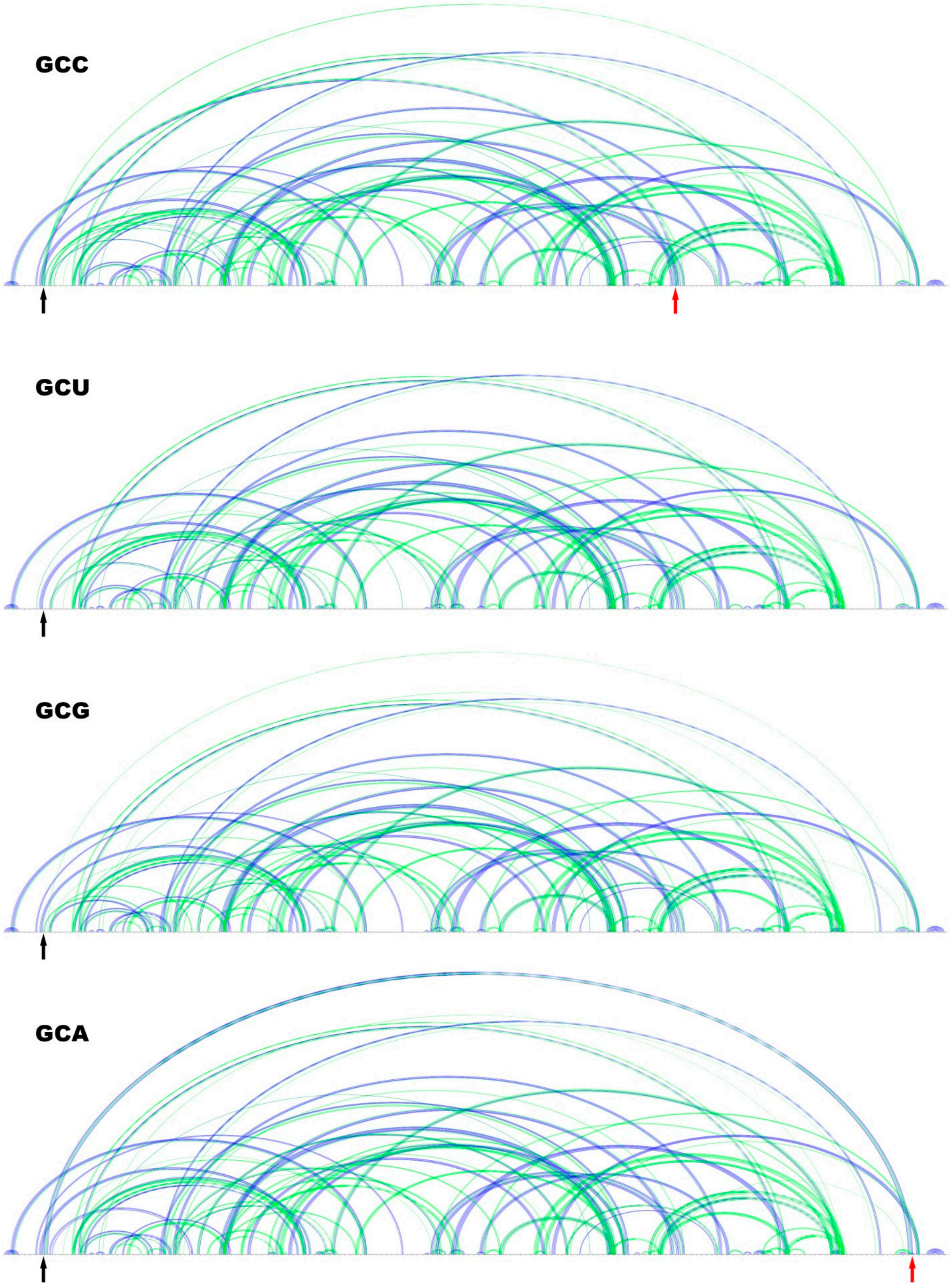

Figure S2. Secondary structures of the CRM197-encoding mRNA variants predicted using SPOT-RNA software. The sequence from the supposed transcription start to the supposed transcription terminator (1825 bases) was taken for the analysis. The nucleotide sequence is shown linear, while the secondary structure is depicted with arcs (blue for canonical base-pairs and green for non-canonical, lone-pairs, and triplets). The mutated codon is written near each structure; its position in the mRNA sequence is shown with a black arrow. The fragment interacting with the mutated codon is shown with a red arrow.

Table S1. The primers used to obtain the CRM197 insertion and deletion mutants

Name	Sequence
AD-GV-FLGC-F	AATCATGATTAAATTTCTCTCTGCATTAATTCTTCTACTGGTCACGACGGCGGCTC AGGCTGGAGCCGACGACGTT
AD-GV-D1-F	ATCCATGGCCGACGACGTTGTTGAC
AD-GV-D2-F	ATCCATGGACGACGTTGTTGACTCTTCT
AD-GV-D3-F	ATCCATGGACGTTGTTGACTCTTCTAAATC
AD-GV-C-R	ATCTCGAGTTATTAAGATTTGATTTCGAAGAAC

Table S2. The primers used to make the point mutations in the CRM197-encoding gene

Name	Sequence
AD-GV-AlaA-F	ATCCATGGGAGCAGACGACGTT
AD-GV-AlaG-F	ATCCATGGGAGCGGACGTTGTT
AD-GV-AlaT-F	ATCCATGGGAGCTGACGTTGTT
AD-GV-Thr-F	ATCCATGGGAACCGACGTTGTT
AD-GV-Pro-F	ATCCATGGGACCCGACGTTGTT
AD-GV-LG-F	ATCCATGGGATCCGACGTTGTT
AD-GV-C-R	ATCTCGAGTTATTAAGATTTGATTTCGAAGAAC

Table S3. Characteristics of the full-length mRNA (1825 bases) calculated using the RNAfold algorithm

Codon	Minimum free energy for the optimal secondary structure, kcal/mol	Frequency of the minimum free energy structure in the ensemble	Ensemble diversity	Free energy of the thermodynamic ensemble, kcal/mol
GCC	–518.60	0.00 %	521.94	–546.13
GCT	–517.60	0.00 %	512.88	–544.92
GCG	–519.70	0.00 %	507.22	–546.55
GCA	–520.20	0.00 %	473.30	–546.82
ACC	–518.90	0.00 %	495.08	–545.76
TCC	–518.40	0.00 %	499.51	–545.28
CCC	–517.50	0.00 %	495.49	–544.72

Table S4. Characteristics of the 140-bases fragment which includes the mutated codon calculated using the RNAfold algorithm

Codon	Minimum free energy for the optimal secondary structure, kcal/mol	Frequency of the minimum free energy structure in the ensemble	Ensemble diversity	Free energy of the thermodynamic ensemble, kcal/mol
GCC	–33.50	1.08 %	17.33	–36.29
GCT	–31.10	0.60 %	21.87	–34.25
GCG	–32.00	0.66 %	26.59	–35.09
GCA	–32.10	0.52 %	27.86	–35.34
ACC	–30.20	0.71 %	22.13	–33.25
TCC	–30.30	0.70 %	23.69	–30.30
CCC	–30.30	0.90 %	20.56	–33.20

Table S5. Characteristics of the 33-bases fragment which includes the mutated codon calculated using the RNAfold algorithm

Codon	Minimum free energy for the optimal secondary structure, kcal/mol	Frequency of the minimum free energy structure in the ensemble	Ensemble diversity	Free energy of the thermodynamic ensemble, kcal/mol
GCC	–7.80	23.81 %	4.57	–8.68
GCT	–5.40	14.01 %	7.17	–6.61
GCG	–6.80	30.90 %	10.65	–7.52
GCA	–7.70	55.12 %	8.14	–8.07
ACC	–4.50	21.19 %	4.80	–5.46
TCC	–4.60	20.26 %	5.07	–5.58
CCC	–4.60	22.89 %	4.09	–5.51
